# Dysregulated lipid metabolism is associated with kidney allograft fibrosis

**DOI:** 10.1186/s12944-024-02021-3

**Published:** 2024-02-03

**Authors:** Linjie Peng, Chang Wang, Shuangjin Yu, Qihao Li, Guobin Wu, Weijie Lai, Jianliang Min, Guodong Chen

**Affiliations:** 1 Organ Transplant Center, The First Affiliated Hospital, Sun Yat-sen University, 58 Zhongshan 2nd Road, Guangzhou, 510080 China; 2 Guangdong Provincial Key Laboratory of Organ Donation and Transplant Immunology, The First Affiliated Hospital, Sun Yat-sen University, Guangzhou, China; 3 The First Affiliated Hospital, Guangdong Provincial International Cooperation Base of Science and Technology (Organ Transplantation), Sun Yat-sen University, Guangzhou, China

**Keywords:** Lipid metabolism, CPT1A, Kidney allograft, Fibrosis, IF/TA

## Abstract

**Background:**

Interstitial fibrosis and tubular atrophy (IF/TA), a histologic feature of kidney allograft destruction, is linked to decreased allograft survival. The role of lipid metabolism is well-acknowledged in the area of chronic kidney diseases; however, its role in kidney allograft fibrosis is still unclarified. In this study, how lipid metabolism contributes to kidney allografts fibrosis was examined.

**Methods:**

A comprehensive bioinformatic comparison between IF/TA and normal kidney allograft in the Gene Expression Omnibus (GEO) database was conducted. Further validations through transcriptome profiling or pathological staining of human recipient biopsy samples and in rat models of kidney transplantation were performed. Additionally, the effects of enhanced lipid metabolism on changes in the fibrotic phenotype induced by TGF-β1 were examined in HK-2 cell.

**Results:**

In-depth analysis of the GEO dataset revealed a notable downregulation of lipid metabolism pathways in human kidney allografts with IF/TA. This decrease was associated with increased level of allograft rejection, inflammatory responses, and epithelial mesenchymal transition (EMT). Pathway enrichment analysis showed the downregulation in mitochondrial LC-fatty acid beta-oxidation, fatty acid beta-oxidation (FAO), and fatty acid biosynthesis. Dysregulated fatty acid metabolism was also observed in biopsy samples from human kidney transplants and in fibrotic rat kidney allografts. Notably, the areas affected by IF/TA had increased immune cell infiltration, during which increased EMT biomarkers and reduced CPT1A expression, a key FAO enzyme, were shown by immunohistochemistry. Moreover, under TGF-β1 induction, activating CPT1A with the compound C75 effectively inhibited migration and EMT process in HK-2 cells.

**Conclusions:**

This study reveal a critical correlation between dysregulated lipid metabolism and kidney allograft fibrosis. Enhancing lipid metabolism with CPT1A agonists could be a therapeutic approach to mitigate kidney allografts fibrosis.

## Background

Kidney transplantation (KT) is currently the first-line and preferred treatment for end-stage chronic kidney disease (CKD), and offers significant improvements in life quality and patient survival. However, chronic renal allograft dysfunction (CAD), is a major concern and poses a threat to the long-term success of the KT [1]. The main histopathological characteristics of CAD include interstitial fibrosis and tubular atrophy (IF/TA), chronic arteriolar alteration, and glomerulosclerosis, as shown by Banff criteria [2, 3]. IF/TA can be detected in over 50% of kidney allografts 2 years after KT [4]. Notably, IF/TA, particularly when coupled with inflammatory cell infiltration, is closely associated with early renal dysfunction, an impaired glomerular filtration rate (GFR), and an increased risk of death-censored kidney allograft failure [5–7]. Despite great advancements in immunosuppressive therapies for early rejection after organ transplantation, currently understanding of the pathophysiology of CAD, particularly IF/TA with or without inflammatory cell infiltration, is still limited [8]. And there is a significant gap in targeted and effective therapeutic interventions.

The transformation of kidney tubular epithelial cells (TECs) into a mesenchymal state, also named epithelial mesenchymal transition (EMT), is increasingly recognized as a critical mechanism in kidney allograft fibrosis [9, 10]. Factors that contribute to IF/TA in kidney allografts include immune-mediated factors, such as humoral rejection and cellular rejection, and nonimmune factors, such as calcineurin inhibitor toxicity (CNIT), the recurrence of native kidney disease, and surgical complications [1, 11]. These factors compromise the repair capabilities of TECs and prompt them to differentiate into mesenchymal cells [12]. Notably, TECs primarily rely on fatty acid β-oxidation (FAO) for energy rather than carbohydrate catabolism [13, 14]. Currently, impaired FAO has now been recognized as a critical pathophysiological mechanism in the process of fibrosis during CKD [15]. Enhancing FAO in TECs has been shown to ameliorate kidney interstitial fibrosis in CKD [16, 17]. However, the significance of lipid metabolism, particularly FAO, in the development of kidney allograft fibrosis has not been fully elucidated.

In this study, the impact of fatty acid metabolism pathway on the development of kidney allograft fibrosis was examined using a combination of public database analysis, clinical biopsy samples, and in vivo and in vitro experiments. Taking lipid metabolism as a breakthrough point, this study was going to shed light on the effects of lipid metabolism in kidney allograft fibrosis, which could help generate new understandings of the molecular underpinnings and inform future treatment approaches.

## Materials and methods

### Ethics statement

This study adhered to the ethical standards of the Declaration of Helsinki and Istanbul. The human kidney tissue in this study was approved by the Ethics Committee of the First Affiliated Hospital of Sun Yat-sen University (ID: 2022 − 166). Written informed consent was obtained from all transplant recipients. Animal handling complied with the guidelines of the United States National Institutes of Health, and was approved from the Sun Yat-sen University Institutional Animal Care and Use Committee (ID: IACUC-2,010,020).

### GEO microarray datasets

Eligible microarray datasets were sourced from the GEO database. Studies that met the whole criteria were considered eligible for further analysis: (1) The studies contained at least 10 patients in both the IF/TA and normal groups; (2) protocol renal allograft biopsy was performed 12 months after kidney transplantation; (3) pathology-verified IF/TA, inflammation within areas of IF/TA (i-IF/TA), or CAD based on the Banff criteria [3]. Studies with other diagnoses, such as humoral rejection, cellular rejection, CNIT, or BK virus infection, were excluded. The datasets included were GSE53605, GSE22459, and GSE76882 (Table 1) [18–20].

**Table 1:** Details of microarrays

Chip serial number	Platform	Normal biopsy	Fibrosis biopsy	Country	Donor	Immunosuppression regimens	Banff	Published year
GSE53605	GPL571	18	10	USA	Deceased	Tarco + MMF + Pred	CAD	2014
GSE22459	GPL1570	25	16	USA	Living	Tarco + MMF + Pred	i-IFTA ≥ 1	2010
GSE76882	GPL13158	99	10	USA	NA	NA	i-IFTA ≥ 1	2016

Tarco, tacrolimus; MMF, mycophenolate mofetil; Pred, prednisone;

### Conversion of raw data and differential expression analysis

We integrated the three datasets using the method of batch normalization with the R package “sva” [21]. Differentially expressed genes (DEGs) were defined as those with a |Log2FC| > 1 and adjusted *P*-value < 0.05 by R package “Limma” [22]. Gene set enrichment analysis (GSEA) [23], Gene Ontology (GO) analysis, Kyoto Encyclopedia of Genes and Genomes (KEGG) analysis, and WikiPathways analysis were used to examine the biological mechanisms. eVITTA (https://tau.cmmt.ubc.ca/eVITTA/) and Metascape (Metascape) were also used for the enrichment analysis [24]. Robust Rank Aggregation (RRA) analysis was performed to identify significantly altered DEGs among three datasets with the R package “Robust Rank Aggreg” [25]. DEGs selected by RRA were then independently enrolled in enrichment analysis.

### Living donor kidney transplant biopsy

The indicated biopsy samples from living donor kidney transplant recipients (2019–2022) at single center were retrospectively assessed. Pathology-verified cases with i-IFTA ≥ 2 at 12 months posttransplantation were considered, and the availability of a zero-time biopsy was also a requirement. A total of 4 recipients with 8 samples in total were enrolled. Slides 3 μm in thickness were prepared from formalin-fixed, paraffin-embedded needle-core biopsies for subsequent immunohistochemical (IHC) analysis, such as hematoxylin and eosin (HE) and Masson’s trichrome staining. Experienced pathologists manually scored the stained slides based on the Banff classification system [26].

### Rat model of kidney allograft fibrosis

Adult male Lewis (LEW/Crl; LEW) and F344 (F344/DuCrl; CDF) inbred rats, aged 8 to 10 weeks and weighing approximately 250 g, were purchased from Charles River Laboratories (Beijing, China).According to a previously described method [27], renal vascular and ureter was anastomosed in an end-to-end manner. In the allograft group, the left kidneys of the F344 rats (donor) were orthotopically transplanted into the left side of Lewis rats (recipient). In the syngeneic group, Lewis rats were used as both donors and recipients. A low dose of tacrolimus (0.1 mg/kg) was administered to the recipients for 7 days to prevent acute rejection, and the recipient’s right kidney was removed on the 7th day after surgery. Fourteen weeks posttransplantation, the rats were euthanized for histological examination and transcriptome profiling.

### In vitro experiment

HK-2 cells (TCH-C400) were obtained from Haixing Biosciences (Suzhou, China) and cultured in high glucose DMEM containing 10% FBS and 1% penicillin‒streptomycin. The cells were maintained at 37 °C in a 5% CO_2_ atmosphere. Briefly, HK2 cells were firstly cultured for 24 h to reach about 80% confluence. New DMEM without FBS were then used for HK-2 starvation overnight before being treated with TGF-β1 (10 ng/ml, ABclonal, China) to induce the EMT process, which mimics the process in kidney fibrosis. The cells in some experiments were pretreated with the Carnitine palmitoyltransferase 1 A (CPT1A) activator C75 (10 µM, HY-12,364, MedChemExpress, USA) or the CPT1A antagonist Etomoxir sodium salt (80 µM, HY-50,202 A, MedChemExpress, USA) for 2 h [28].

### Immunohistochemical and immunofluorescence analysis

Sections from both rats and humans were incubated in room temperature with antibodies against α-SMA (1:100, ABclonal, China), E-cadherin (1:100, ABclonal, China) and CPT1A (1:100, ABclonal, China). The sections were then incubated with HRP linked secondary antibody and stained using ZSGB-BIO (Beijing, China). Immunofluorescence analysis were performed with primary antibodies against CPT1A (1:100; ABclonal, China), α-SMA (1:100; ABclonal, China), and FN (1:100; ABclonal, China). Digital images were captured using ECLIPSE 80i microscope (Nikon, Japan). IHC linear measurements and the fluorescence intensities were performed using ImageJ software 1.46r (National Institutes of Health, U.S.A.).

### **Real-time quantitative polymerase chain reaction**

Total RNA was extracted from the rat kidneys with TRIzol reagent according to the manufacturer’s instructions (Thermo Fisher Scientific, USA). One microgram of RNA was reverse transcribed with the qScript cDNA Synthesis kit (Quanta Biosciences, Gaithersburg, MD). The real-time quantitative PCR (RT‒qPCR) was performed in a 15 µl of SYBR Green reaction mix (Bio-Rad) containing 40 ng cDNA template and 10 nM primers. Bio-Rad IQ2 PCR instrument were used. Gene expression levels were normalized to those of the control gene β-actin. Alters of gene expression levels were calculated by the 2^−ΔCt^ method. The specific primers used in rat are showed in Table 2.

**Table 2:** Primer sequences used for RT-qPCR in rat

Target Gene	Forward primer (5’-3’)	Reverse primer (5’-3’)
ACTA (α-SMA)	CATCCGACCTTGCTAACGGA	AATAGCCACGCTCAGTCAGG
Vimentin	TCAGACAGGATGTTGACAAT	GACATGCTGTTCCTGAATCT
CPT1A	ACCCAGTCAGATTCCAACCA	TCAAGAAAGGCACTCCCACT
Actinb	GCCTTCCTTCCTGGGTATGG	AATAGCCACGCTCAGTCAGG

### Statistical analysis

Data analysis was performed using R software (GraphPad Software, Inc., La Jolla, CA, USA) and GraphPad Prism (GraphPad Software, Inc., San Diego, CA). To determine statistical significance of differences between two groups, an unpaired t-test was used. For data involving more than two groups, one-way ANOVA was used. A *P*-value less than 0.05 was considered to indicate statistical significance.

## Results

**Fig. 1:**
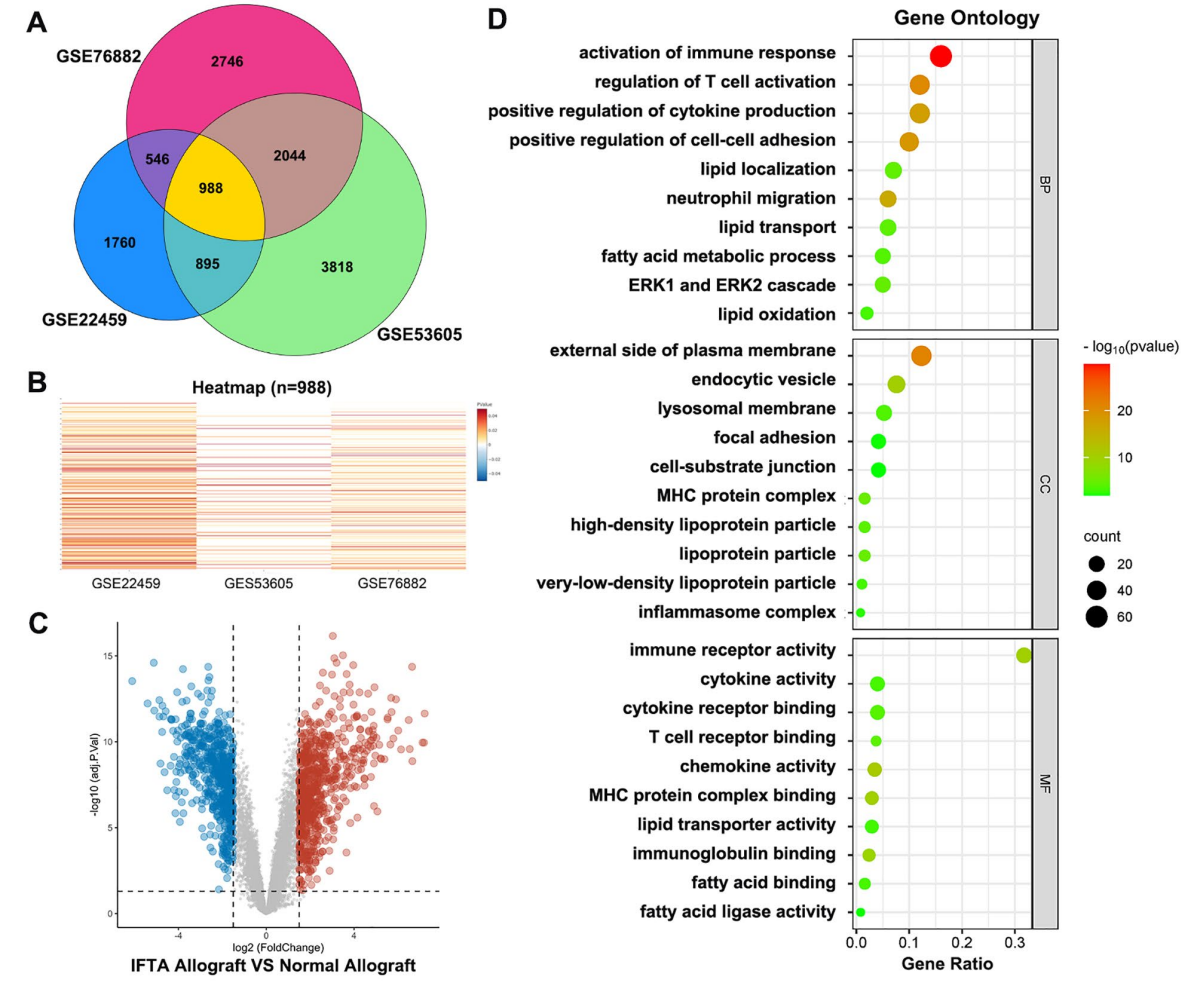
Data integration of three GEO datasets and GO enrichment analysis. **(a)** Venn diagram showing 988 coexpressed genes among the three GEO datasets. **(b)** Heatmap showing the expression of 988 coexpressed genes from the three GEO datasets. **(c)** Volcano plot showing 135 downregulated genes and 560 upregulated genes after data integration. **(d)** Gene Ontology analysis of biological processes, cellular components and molecular functions

### In-depth analysis of the GEO database revealed the key role of the lipid metabolism pathway in human kidney allograft fibrosis

Three datasets from the GEO database, including 50 samples from human kidney allografts with IF/TA and 142 samples from normal transplanted kidneys, were used for the integrated analysis (Table 1). Heatmap and Venn diagram analyses identified 988 coexpressed genes among the three datasets (Fig. 1A and B). A total of 695 DEGs were found, including 135 downregulated genes and 560 upregulated genes, as showed in the volcano plot (Fig. 1C). Gene Ontology (GO) analysis revealed significant enrichment in 882 biological processes (BPs), 80 cellular components (CCs), and 120 molecular functions (MFs). The predominant biological processes included immune response activation, T‒cell activation regulation, and regulation of cell‒cell adhesion, while lipid localization, lipid transport, and lipid oxidation were also functionally enriched. MHC protein complexes, focal adhesions, and lipoprotein particles were enriched among CCs. Immune receptor activity, cytokine activity, as well as lipid transporter activity were enriched among MFs **(**Fig. 1D). These results indicated that immune cell participation, focal adhesion changes, and lipid metabolism-related events were closely associated in kidney allograft fibrosis.

**Fig. 2:**
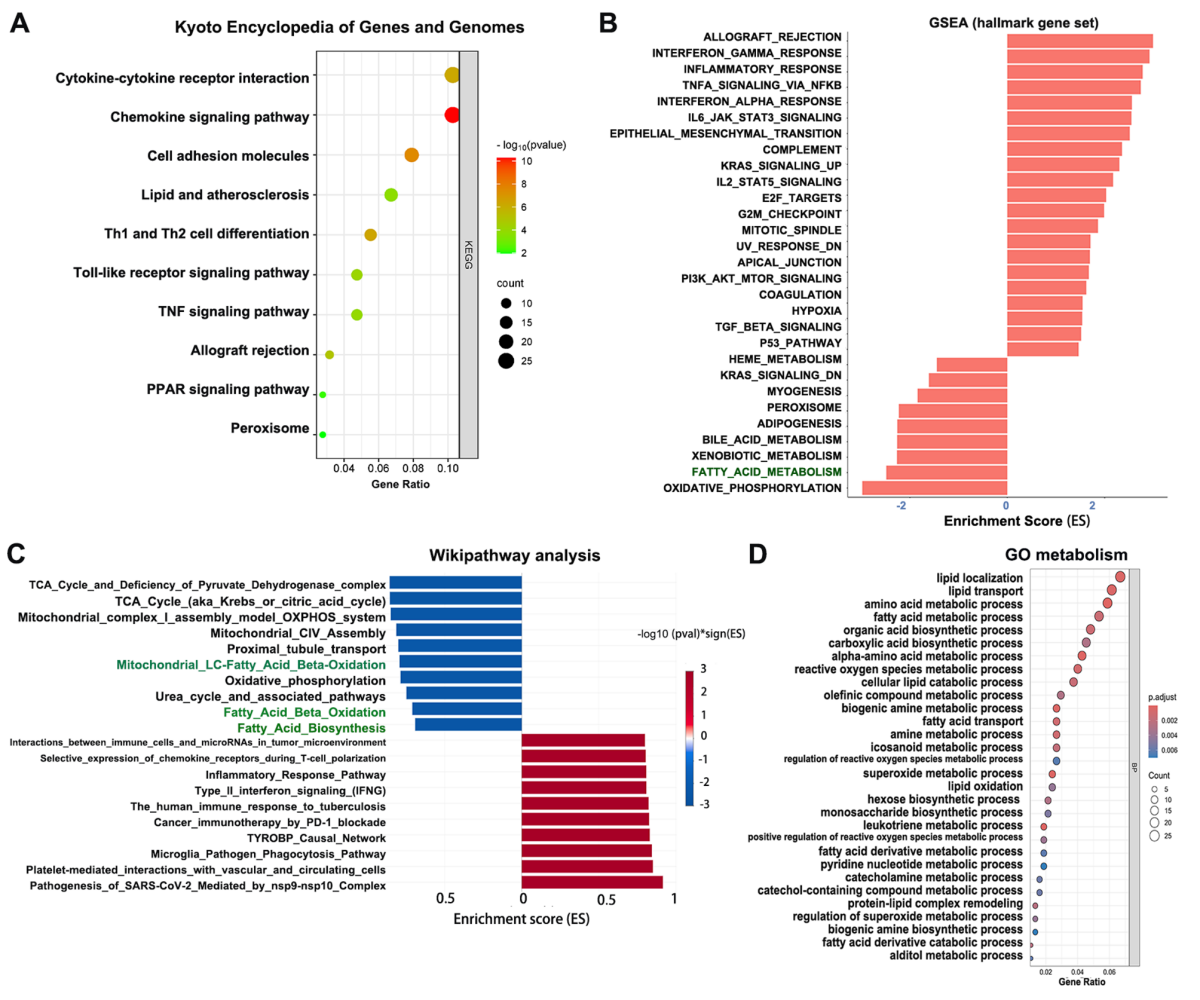
Enrichment analysis of the GEO data. **(a)** Kyoto Encyclopedia of Genes and Genomes pathway analysis. **(b)** Gene set enrichment analysis using the hallmark gene set. **(c)** WikiPathwayays enrichment analysis. **(d)** GO metabolic analysis

KEGG pathway analysis revealed enrichment of immune-related pathways, cell adhesion molecules, lipid and atherosclerosis, and the peroxisome proliferator-activated receptor (PPAR) signaling pathway (Fig. 2A). Gene Set Enrichment analysis (GSEA) and WikiPathways enrichment analysis consistently showed the downregulation of energy metabolism pathways, such as mitochondrial LC-fatty acid beta-oxidation, FAO and fatty acid biosynthesis in kidney allograft fibrosis. Moreover, the activation of allograft rejection, the inflammatory response pathway, and the EMT pathway were significantly upregulated during kidney allograft fibrosis (Fig. 2B-D). The interplay between lipid metabolism, the immune response, and EMT plays a critical role in renal allograft fibrosis. Regarding metabolic processes in the kidney allograft, GO annotation showed the predominance of lipid-related metabolic processes, such as lipid localization, lipid transport, fatty acid metabolic processes, and lipid catabolic processes (Fig. 2C). These finding indicated that the downregulation of lipid metabolism could further impair the repair capacity of TECs during kidney allograft fibrosis.

**Fig. 3:**
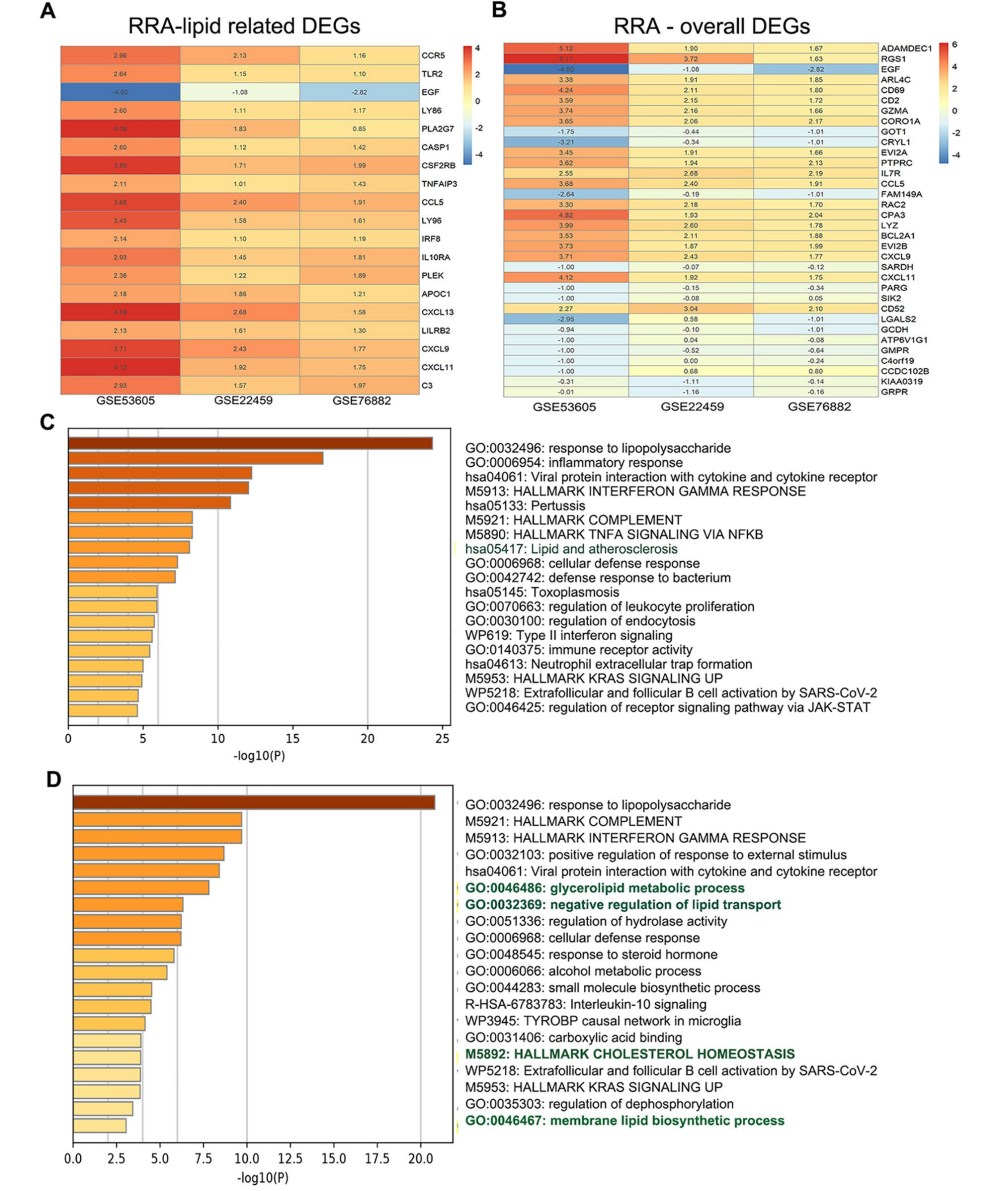
Robust rank aggregation analysis and enrichment analysis. **(a)** Lipid metabolism related-DEGs among the three datasets ranked by Robust rank aggregation analysis. **(b)** Overall DEGs across three datasets ranked by Robust Rank Aggregation analysis. **(c)** Enrichment analysis of lipid metabolism related-DEGs showed in Fig. 3A. **(d)** Enrichment analysis of DEGs shown in Fig. 3B

Robust Rank Aggregation (RRA) analysis identified significant DEGs among the datasets. Key lipid-related DEGs, such as CCR5, CCL5, and TLR2, were upregulated, indicating their association with inflammatory responses in kidney allograft fibrosis (Fig. 3A). EGF was the most significantly downregulated gene, suggesting its potential as a biomarker for lipid metabolism downregulation in this context (Fig. 3A). Similar results were observed for lipid metabolism and inflammatory cell infiltration among the overall ranked significant DEGs in the kidney allograft fibrosis (Fig. 3B). Enrichment analysis of these DEGs also highlighted pathways involved in lipid metabolism and inflammatory cell infiltration (Fig. 3C and D).

**Fig. 4:**
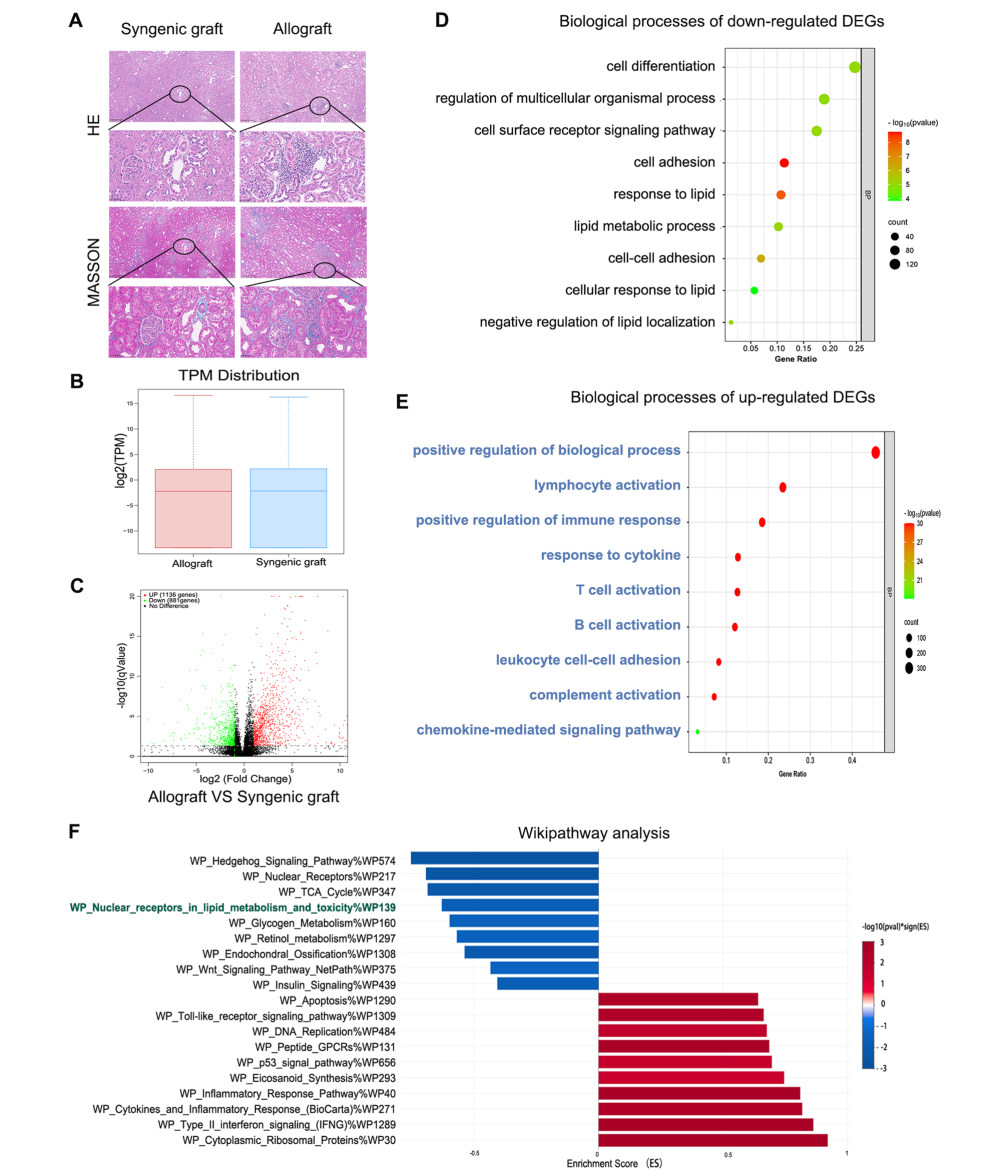
Pathological staining and transcriptome profiling analysis in rat model of kidney transplantation. **(a)** HE and Masson staining showed extensive mononuclear cell infiltration and an increased fibrosis area, respectively, in the allograft group. **(b)** The transcript per million (TPM) distribution shows the eligible transcriptome data between the allograft group (fibrosis) and the syngeneic allograft group (normal). **(c)** Volcano plot showing 1136 upregulated genes and 881 downregulated genes compared to those in the syngeneic allograft group. **(d)** GO analysis of the 1136 up regulated DEGs. **(e)** GO analysis of the 881 downregulated DEGs. **(f)** WikiPathways enrichment analysis of all 2017 DEGs

### Downregulated fatty acid metabolism in rat kidney allografts with fibrosis

To further validate the findings from the GEO dataset, a rat models of kidney transplant with a low-dose immunosuppression was performed. The kidney grafts (allografts: the fibrosis group, *n* = 7 versus syngeneic graft: the normal group, *n* = 6) were harvested at 14 weeks posttransplantation. As expected, extensive inflammatory cell infiltration in the local IF/TA area (i-IF/TA) was observed in rat kidney allografts but not in syngeneic grafts, as indicated by HE and Masson staining (Fig. 4A). Transcriptome profiling of the kidney grafts revealed a similar TPM distributions between the fibrosis group and the normal group (Fig. 4B), with 1136 upregulated genes and 881 downregulated genes (Fig. 4C). GO analysis of the downregulated genes showed pathways related to cell differentiation, cell adhesion, and lipid metabolism, suggesting their role in EMT and kidney allograft fibrosis (Fig. 4D). GO analysis of the upregulated genes revealed the activation of immunological responsive cells such as lymphocytes, T lymphocyte and B lymphocyte, as well as cytokines, complement and chemokines (Fig. 4E). WikiPathways analysis confirmed the significant downregulation of lipid metabolism and the enrichment of immune response pathways (Fig. 4F).

**Fig. 5:**
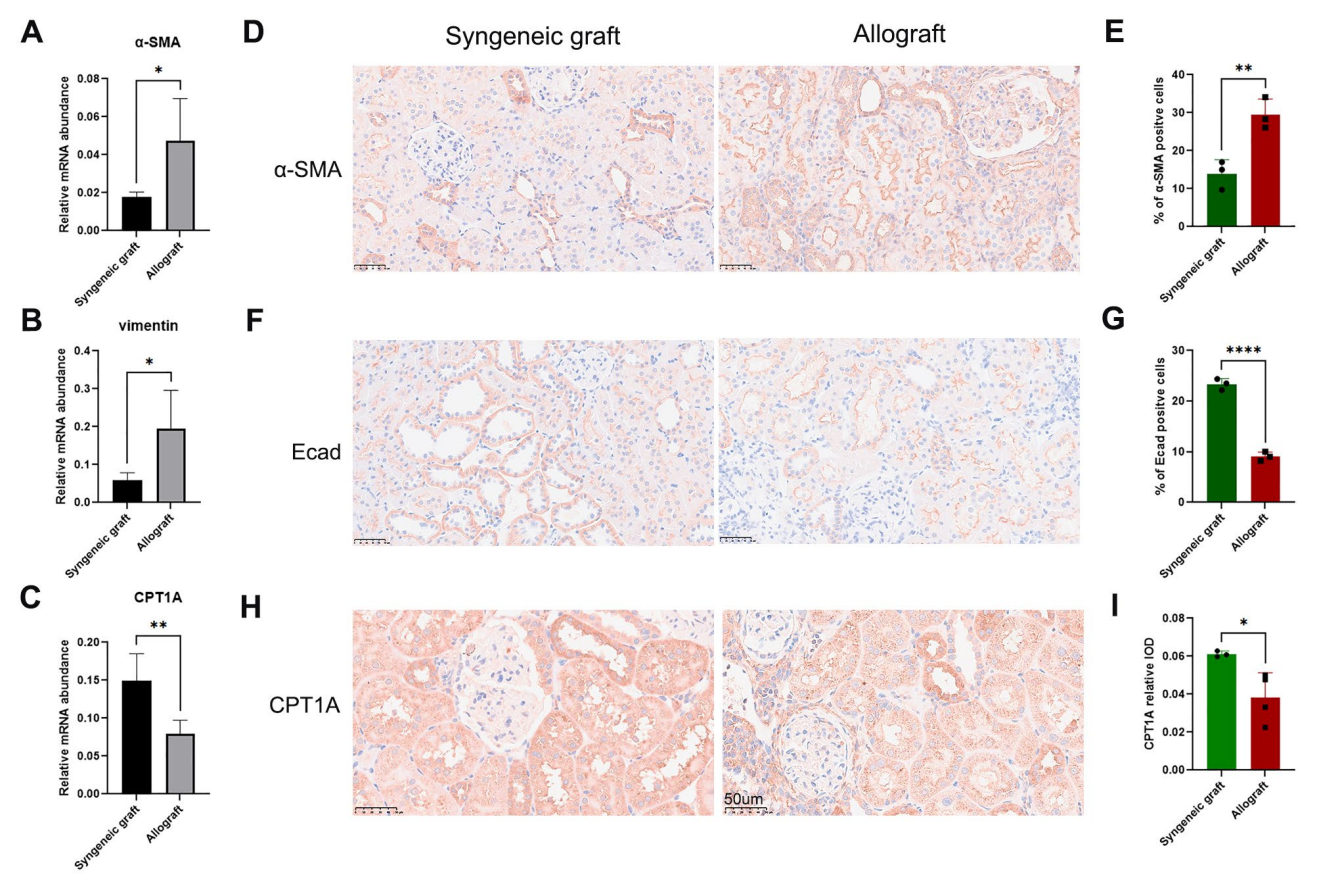
Immunohistochemical analysis of EMT and CPT1A expression in a rat model of kidney transplantation. **(a)** The gene expression level of α-SMA in the indicated kidney tissues was analyzed by RT-qPCR. **(b)** The gene expression level of vimentin in the indicated kidney tissues was analyzed by RT-qPCR. **(c)** The gene expression level of CPT1A in the indicated kidney tissues was analyzed by RT-qPCR. **(d)** Representative IHC image of α-SMA. **(e)** The percentages of α-SMA-positive cells were calculated. **(f)** Representative IHC image of E-cadherin. **(g)** The percentages of E-cadherin-positive cells were calculated. **(h)** Representative IHC image of CPT1A. **(i)** The intensity of CPT1A expression was calculated. * *P* < 0.05, ** *P* < 0.01, **** *P* < 0.0001, unpaired t test

**Fig. 6:**
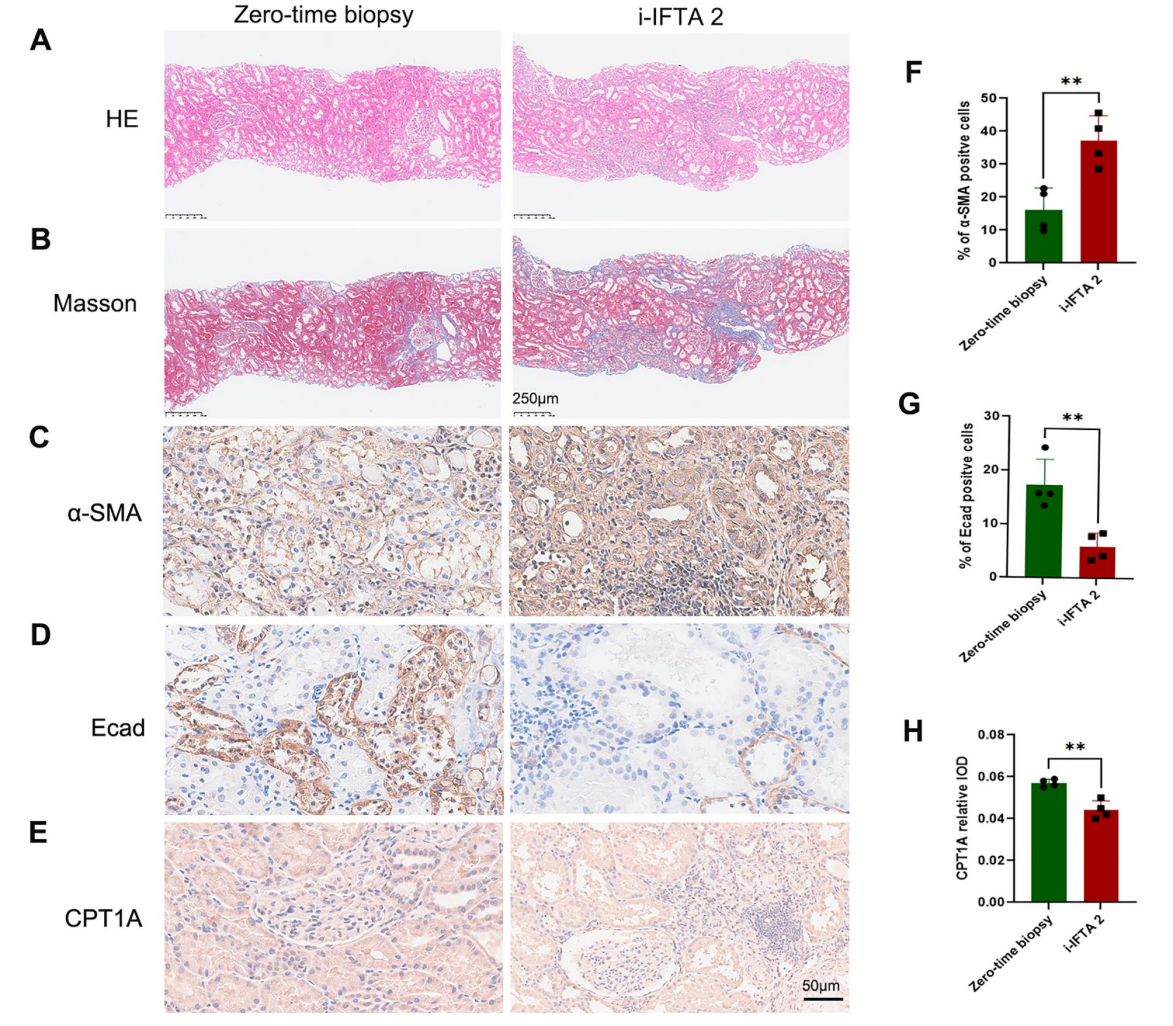
Pathological staining and Immunohistochemical analysis of EMT and CPT1A expression in human kidney allograft fibrosis. **(a)** Representative HE staining. **(b)** Representative Trichrome-Masson staining. **(c)** Representative IHC image of α-SMA. **(d)** Representative IHC image of E-cadherin. **(e)** Representative IHC image of CPT1A. **(f)** The percentages of α-SMA-positive cells were calculated. **(g)** The percentages of E-cadherin-positive cells were calculated. **(h)** The intensity of CPT1A expression was calculated. ** *P* < 0.01, unpaired t test

### Reduced expression of CPT1A was observed in both rat and human kidney allografts with fibrosis

In rat kidney transplants, RT-qPCR showed high levels of α-SMA and vimentin expression (Fig. 5A and B). IHC analysis confirmed the increase in α-SMA and decrease in E-cadherin expression in i-IF/TA areas, suggesting the involvement of EMT in kidney allograft fibrosis (Fig. 5D, E and F, and 5G). CPT1A is a key enzyme in FAO. Surprisingly, the levels of CPT1A expression were significantly reduced in i-IFTA areas (Fig. 5H and I), as showed by RT-qPCR (Fig. 5C).

In human kidney allograft biopsy samples, extensive inflammatory cell infiltration was found in the IF/TA area (i-IF/TA score ≥ 2) (Fig. 6A), and evident fibrosis was detected by Masson staining (Fig. 6B). IHC staining revealed increased expression of α-SMA (Fig. 6C and F) and decreased expression of E-cadherin (Fig. 6D and G), which was consistent with the changes in the expression of EMT markers in the rat model, highlighting the involvement of EMT in kidney allograft fibrosis. In addition, the expression of CPT1A was reduced in TECs around the i-IFTA area (Fig. 6E and H), mirroring the findings in the rat kidney transplant models. These results underscore the close connection between EMT and lipid metabolism, suggesting that pharmacologically targeting CPT1A could be a therapeutic strategy for prevention or treatment of kidney allograft fibrosis.

**Fig. 7:**
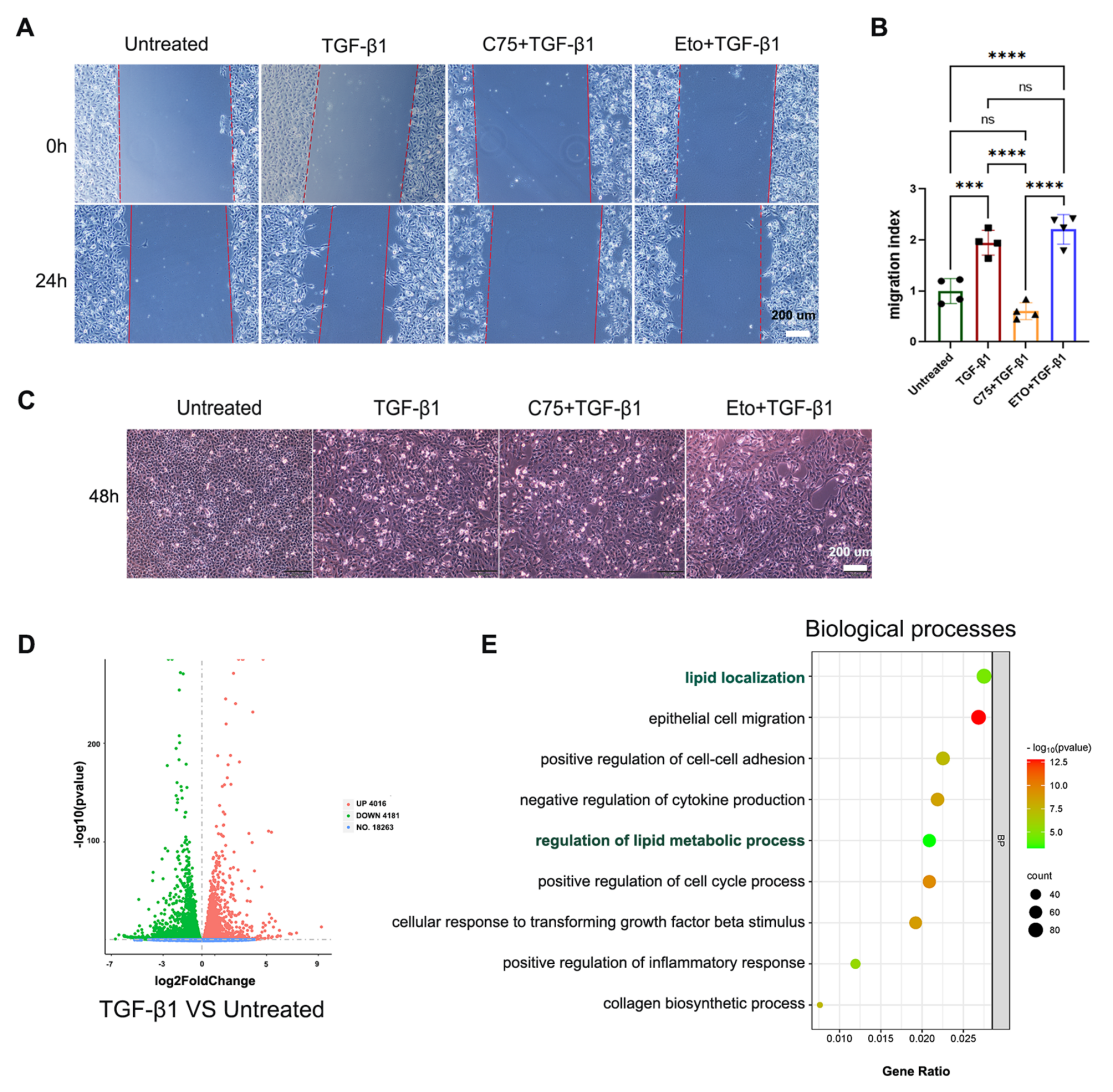
In vitro model of EMT induced by TGF-β1. **(a)** A scratch healing assay showed that C75 could inhibit HK-2 cell migration induced by TGF-β1 at 24 h. **(b)** Comparison of migration indices among the four intervention groups. The migration index is defined as maximum migration distance at 24 h; *** *P* < 0.001, **** *P* < 0.0001; one-way ANOVA. **(c)** Changes in HK-2 cell morphology at 48 h in four intervention groups. **(d)** Volcano plot showing the DEGs between untreated group and TGF-β1 group. **(e)** GO analysis of the DEGs shown in Fig. 7D

### Activating CPT1A reduced the EMT process in tubular cells in vitro

According to the result of the cell scratch assays, CPT1A agonists (C75) significantly inhibited the migration of HK-2 cells at 24 h. In contrast, the TGF-β1 and TGF-β1 + Eto (CPT1A antagonists) group exhibited increased migration rates (Fig. 7A and B). Morphological analysis at 48 h after TGF-β1 or TGF-β1 + Eto treatment revealed distinct changes in cell shape, in contrast to the preserved morphology of C75-treated cells (Fig. 7C). These data suggest that C75 is effective at mitigating EMT process in tubular cells. Furthermore, RNAseq analysis of murine TECs treated with TGF-β1 identified more than 8000 DEGs (Fig. 7D), and the GO terms were enriched in lipid metabolism, epithelial cell migration, and cell-cell adhesion pathways (Fig. 7E).

**Fig. 8:**
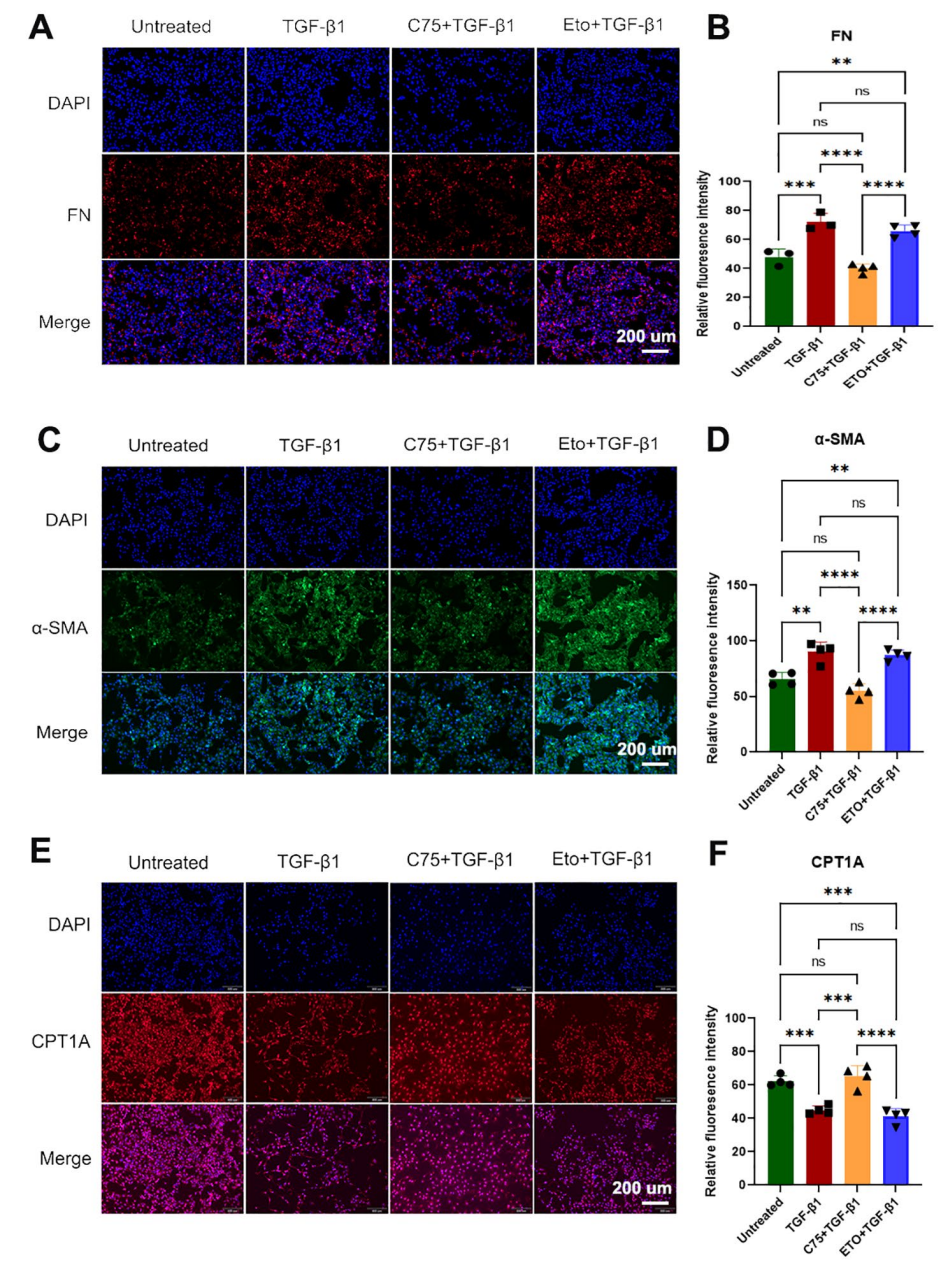
Immunofluorescence analysis of EMT and CPT1A expression in the in vitro model. **(a)** Immunofluorescence analysis of fibronectin expression. **(b)** The relative fluorescence intensity of fibronectin in Fig. 8A was determined by ImageJ. **(c)** Immunofluorescence analysis of α-SMA expression. **(d)** The relative fluorescence intensity of α-SMA in Fig. 8C was determined by ImageJ. **(e)** Immunofluorescence assay of CPT1A expression. **(F)** The relative fluorescence intensity of α-SMA in Fig. 8E was determined by ImageJ. ** *P* < 0.01, *** *P* < 0.001, **** *P* < 0.0001, one-way ANOVA

According to the immunofluorescence assay, both TGF-β1 and ETO + TGF-β1 significantly increased the expression of FN and α-SMA (Fig. 8A-D), confirming the occurrence of EMT. C75 increased CPT1A expression and decreased FN and α-SMA expression (Fig. 8A-D), which was comparable to that in the untreated group (Fig. 8E and F). Additionally, blocking CPT1A with ETO significantly decreased the expression of CPT1A, which was comparable to the effect of TGF-β1 alone (Fig. 8F). This in vitro model supports the therapeutic potential of CPT1A activation for alleviating EMT and renal fibrosis.

## Discussion

This present study revealed a significant downregulation of lipid metabolism in kidney allografts with fibrosis, as evidenced by the integrated analysis of GEO datasets, transcriptomic analysis and immunohistochemical (IHC) staining of human biopsies one year after kidney transplantation and in rat models. This downregulation in lipid metabolism was closely correlated with the epithelial mesenchymal transition (EMT) and the activation of immune cells. Histological evaluations, including HE, Masson, and IHC staining, further validated these transcriptomic findings in both living-donor kidney transplantation and rat transplant models. Notably, activating CPT1A, a crucial enzyme in FAO, inhibited the migration of HK-2 cells and mitigated the expression of EMT biomarkers, indicating the involvement of lipid metabolism downregulation in kidney allograft fibrosis and suggesting that CPT1A activation could be a therapeutic strategy for prevention or treatment.

Chronic inflammation and IF/TA are widely recognized as pivotal indicators of CAD [29]. In particular, when IF/TA is accompanied by inflammatory cell infiltration, recipients face an increased risk of early kidney allograft dysfunction, impaired GFR and death-censored graft failure [5–7]. The cumulative damage, stemming from both immune and nonimmune factors, impairs the regenerative capacity of TECs, prompting their transition into fibroblasts and myofibroblasts [30, 31]. Myofibroblasts can arise from various cell types, including TECs [32]. As part of the EMT process, damaged TECs lose some epithelial markers such as E-cadherin and acquire mesenchymal features like α-SMA [33]. The present study reinforced the pivotal role of EMT in kidney allograft fibrosis, as evidenced by both GO and KEGG pathway analyses highlighted significant alterations in cell adhesion. The upregulation of EMT pathway was identified as one of the most prominent pathways by gene set enrichment analysis. Furthermore, the increase in α-SMA expression and reduce in E-cadherin expression in kidney allografts from human and rats showed the importance of EMT in kidney allograft fibrosis. However, effective therapeutic strategies to reverse EMT are limited.

As we known, the kidney is an organ with high mitochondrial content and substantial energy demands. However, further investigations of the role of energy metabolism in EMT and kidney allograft fibrosis are needed [34]. Under normal conditions, glomeruli prefer glucose, while TECs predominantly rely on fatty acids for energy [35]. Fatty acid oxidation (FAO) is the preferred energy source for highly metabolic TECs, rendering them susceptible to lipid accumulation [13, 35]. Lipid accumulation in TECs triggers inflammation, remodels the actin cytoskeleton, and ultimately leads to cell death [15]. On the other hand, metabolic complications including obesity, insulin resistance (IR) and dyslipidemia are commonly found following kidney transplantation and are associated with intragraft macrophage infiltration and progressive kidney fibrosis [36]. Finding in the present study are consistent with previous findings in nontransplant models, such as the unilateral ureteral obstruction (UUO) mouse model, which also indicates significant alterations in genes related to fatty acid oxidation [35, 37–39].

The present investigation further showed the downregulation of lipid metabolism pathways, including FAO, biosynthesis, and transport pathways, which are closely linked to an increase in immune cell activation. This indicates the disruption of lipid metabolism during kidney allograft fibrosis. Prior researches have established a link between reduced FAO enzyme expression, increased intracellular lipid deposition, and exacerbated TEC damage [37], which is consistent with results from single-cell nuclei RNA sequencing analysis of human kidney allografts [40]. Reduced expression of FAO enzymes may lead to increased intracellular lipid deposition and worsened TEC damage [41]. Previous studies have shown that AMPK and PPAR signaling pathways are valuable targets for treating kidney fibrosis [37]. Consequently, we investigated the expression of the key FAO enzyme CPT1A, as it serves as the rate-limiting enzyme for long acyl-CoA chains entering into the mitochondrial matrix [42]. In the present study, the decreased expression of CPT1A in rat fibrotic kidney allografts, which was confirmed by IHC analysis, indicates its potential as a therapeutic target. In vitro, HK-2 cells exhibited a positive regulation of the cell cycle in response to TGF-β1 induction, and C75-mediated CPT1A activation effectively inhibited EMT, as evidenced by reductions in cell migration and α-SMA expression. Previous studies have shown that CPT1A overexpression owns antifibrotic effects, reduces inflammatory responses, and mitigates epithelial cell damage, reinforcing its therapeutic potential in kidney allograft fibrosis [35].

### Study strengths and limitations

This study presents novel findings that dysregulated lipid metabolism is associated with kidney allograft fibrosis. It explores the role of lipid metabolism in the process of renal allograft fibrosis across comprehensive data, including transcriptomic profiling of human kidney allograft and rat kidney transplantation, pathological staining, IHC and immunofluorescence. These findings propose that the key FAO enzyme - CPT1A holds promise as potential target to prevent or treat kidney allograft fibrosis in the clinical practice. Nevertheless, a notable limitation of the present study is the absence of animal validation of the CPT1A agonist C75. Currently, no specific drugs can activate CPT1A in the clinic, indicating that pharmacological studies have a long way to go. Additionally, the correlation between mononuclear cell infiltration and lipid metabolism in TECs was not discussed in the present study, because the present study mainly focused on lipid metabolism in TECs and development of IFTA, which was consistent with the design of an in vitro model using HK-2 cells.

## Conclusions

The present study highlights the significant link between the reduction in lipid metabolism and the development of kidney allograft fibrosis through the analysis of GEO database entries, clinical kidney allograft samples, and a rat model of kidney transplant and in vitro experiments. Moreover, the modulation of CPT1A has emerged as a promising strategy for enhancing lipid metabolism in tubular epithelial cells, demonstrating its potential as an antifibrotic strategy in kidney transplantation.

## Data Availability

Data is provided within the manuscript or supplementary information files.

## Abbreviations

CKD: Chronic kidney disease
KT: Kidney transplantation
CAD: Chronic renal allograft dysfunction
IF/TA: Interstitial fibrosis and tubular atrophy
EMT: Epithelial mesenchymal transition
CNIT: Calcineurin inhibitor toxicity
i-IFTA: Inflammation within areas of interstitial fibrosis and tubular atrophy
CPT1A: Carnitine palmitoyltransferase 1 A
FAO: Fatty acid β-Oxidation
TEC: Tubular epithelial cells
GEO: Gene Expression Omnibus
DEGs: Differentially expressed genes
GSEA: Gene set enrichment analysis
DEGs: Differentially expressed genes
GO: Gene Ontology
KEGG: Kyoto Encyclopedia of Genes and Genomes
IHC: Immunohistochemical analysis
HE: Hematoxylin and eosin
RT-qPCR: real-time quantitative PCR
